# The Apical Localization of Na^+^, K^+^-ATPase in Cultured Human Retinal Pigment Epithelial Cells Depends on Expression of the β_2_ Subunit

**DOI:** 10.3389/fphys.2016.00450

**Published:** 2016-10-07

**Authors:** Jorge A. Lobato-Álvarez, María L. Roldán, Teresa del Carmen López-Murillo, Ricardo González-Ramírez, José Bonilla-Delgado, Liora Shoshani

**Affiliations:** ^1^Laboratory of Epithelial Research, Department of Physiology, Biophysics and Neurosciences, CINVESTAV-IPNMéxico City, Mexico; ^2^Department of Molecular Biology and Histocompatibility, Hospital General Dr. Manuel Gea GonzálezMéxico City, Mexico; ^3^Research Unit, Laboratory of Genetics and Molecular Diagnosis, Hospital Juárez de MéxicoMéxico City, Mexico

**Keywords:** Na^+^, K^+^-ATPase, retinal pigment epithelium, apical polarity, ARPE-19, AMOG/β_2_, re-morphogenesis

## Abstract

Na^+^, K^+^-ATPase, or the Na^+^ pump, is a key component in the maintenance of the epithelial phenotype. In most epithelia, the pump is located in the basolateral domain. Studies from our laboratory have shown that the β_1_ subunit of Na^+^, K^+^-ATPase plays an important role in this mechanism because homotypic β_1_-β_1_ interactions between neighboring cells stabilize the pump in the lateral membrane. However, in the retinal pigment epithelium (RPE), the Na^+^ pump is located in the apical domain. The mechanism of polarization in this epithelium is unclear. We hypothesized that the apical polarization of the pump in RPE cells depends on the expression of its β_2_ subunit. ARPE-19 cells cultured for up to 8 weeks on inserts did not polarize, and Na^+^, K^+^-ATPase was expressed in the basolateral membrane. In the presence of insulin, transferrin and selenic acid (ITS), ARPE-19 cells cultured for 4 weeks acquired an RPE phenotype, and the Na^+^ pump was visible in the apical domain. Under these conditions, Western blot analysis was employed to detect the β_2_ isoform and immunofluorescence analysis revealed an apparent apical distribution of the β_2_ subunit. qPCR results showed a time-dependent increase in the level of β_2_ isoform mRNA, suggesting regulation at the transcriptional level. Moreover, silencing the expression of the β_2_ isoform in ARPE-19 cells resulted in a decrease in the apical localization of the pump, as assessed by the mislocalization of the α_2_ subunit in that domain. Our results demonstrate that the apical polarization of Na^+^, K^+^-ATPase in RPE cells depends on the expression of the β_2_ subunit.

## Introduction

Na^+^, K^+^-ATPase, or the Na^+^ pump, is the principal transporter in eukaryotic cells that sustains a non-equilibrium distribution of Na^+^ and K^+^ ions across the plasma membrane (Kaplan, 2002). Na^+^, K^+^-ATPase is a heterodimer that consists primarily of α and β subunits. The α subunit has a molecular mass of 110 kDa and is responsible for the catalytic functions of the enzyme (Ohtsubo et al., 1990). The β subunit is a glycoprotein with a molecular mass of 35 kDa and is indispensable for the structural stabilization and functional maturation of the holoenzyme (Geering et al., 1989; Ackermann and Geering, 1990) and the transport of the α subunit to the plasma membrane (Noguchi et al., 1987; Martin-Vasallo et al., 1989). Ion transport requires the participation of both α and β subunits (Fambrough, 1988; Martin-Vasallo et al., 1989). There are four distinct isoforms of the α subunit (α_1_, α_2_, α_3_, and α_4_) and three isoforms of the β subunit (β_1_, β_2_, and β_3_) that are tissue-specific in their expression (Fambrough, 1988; Cortas et al., 1991; Blanco and Mercer, 1998). Finally, there is a small γ subunit that belongs to the FXYD family of proteins that modulates Na^+^, K^+^-ATPase activity (Cortas et al., 1991).

The establishment of cell surface polarity for most membrane proteins in epithelia implicates sorting signals that are encoded in their amino acid sequence (Sweadner et al., 2000; Rodriguez-Boulan et al., 2005), trafficking routes that involve apical or basolateral recycling endosomes (Weisz and Rodriguez-Boulan, 2009), and interactions with epithelial-specific protein complexes such as AP-1B and clathrin, which may be regulated by small GTPases (Ellis et al., 2006; Gonzalez and Rodriguez-Boulan, 2009; Weisz and Rodriguez-Boulan, 2009). Na^+^, K^+^-ATPase is polarized and directed toward the basolateral membrane of most epithelial cells (Deborde et al., 2008) and, more specifically, at cell borders facing the intercellular space (Contreras et al., 1995; Cereijido et al., 2001). In epithelial cells, newly synthesized Na^+^, K^+^-ATPase is delivered directly to the basolateral membrane (Contreras et al., 1989; Shoshani et al., 2005). Although it is clear that the α_1_ subunit carries the information for the basolateral targeting of Na^+^, K^+^-ATPase in typical epithelia (Mays et al., 1995), efforts to identify an amino acid sequence that functions as a basolateral polarity signal in the α_1_ subunit have been unsuccessful (Dunbar et al., 2000). In the target membrane domain, the asymmetric distribution of Na^+^, K^+^-ATPase is reinforced by selective retention through binding to the ankyrin-fodrin cytoskeleton (Hammerton et al., 1991; Muth et al., 1998). Several lines of evidence have demonstrated that the β_1_ subunit anchors the pump at the lateral borders of epithelial cells through homotypic β_1_-β_1_ interactions, provided the neighboring cells express an identical β_1_ subunit (Contreras et al., 1995; Shoshani et al., 2005). Recent studies have further shown that the adhesive properties of the β_1_ subunit play a principal role in the basolateral localization of the pump (Vagin et al., 2006; Padilla-Benavides et al., 2010). However, in the choroid plexus epithelium (Wright, 1972), cockroach salivary gland epithelium (Just and Walz, 1994) and retinal pigment epithelium (RPE; Gundersen et al., 1991), Na^+^, K^+^-ATPase is expressed in the apical membrane.

The RPE makes up the outmost layer of the retina and has many supporting functions that are fundamental for the survival of photoreceptors. The RPE forms the outer hemato-retinal barrier and regulates the volume and chemical composition of the subretinal space. Na^+^, K^+^-ATPase is vital for several RPE cell functions, such as the vectorial transport of ions and solutes from the choroid to the photoreceptors and the reestablishment of Na^+^ and K^+^ gradients required for the photoreceptor dark current, synaptic activity, action potentials, and transmitter uptake in the subretinal space (Miller and Steinberg, 1979). RPE cells are distinctive in that they contain apical Na^+^, K^+^-ATPase (Miller and Steinberg, 1979; Gundersen et al., 1991). Nevertheless, depending on the RPE preparation studied, apical expression can be lost (Geisen et al., 2006) or accompanied by basolateral expression (Okami et al., 1990; Hu et al., 1994; Marrs et al., 1995). Despite many years of investigation, the sorting signals and mechanisms that mediate the apical polarization of Na^+^, K^+^-ATPase remain poorly understood (Cereijido et al., 2012).

The present work focuses on the intriguing mechanism underlying the polarity of the Na^+^ pump in the RPE. Because the β_1_ subunit plays a key role in the basolateral localization of the pump in classic epithelia, we anticipated that β subunit isoforms may be crucial elements in explaining the apical localization of the pump in the RPE. In this context, it is worth recalling that a role for the β subunit, particularly the β_2_ isoform, in the apical polarization of Na^+^, K^+^-ATPase has been suggested in previous studies (Wilson et al., 2000; Vagin et al., 2005). In the present study, we examined the hypothesis that the apical targeting of Na^+^, K^+^-ATPase in RPE cells depends on the expression of the β_2_ subunit.

## Materials and methods

### Reagents and antibodies

The following reagents were used: DMEM, F12, PBS, and FBS (GIBCO Cat. 12100-061, Cat. 21700-026, Cat. 21300-058, and Cat. A15-751), the antibiotics penicillin and streptomycin (10,000 U/μg/ml, *In vitro*, A-01), laminin (SIGMA-ALDRICH Cat. L2020), ITS (a mixture of insulin, human transferrin and selenic acid, BD Biosciences Cat. 354352), Protease Inhibitor Mix (GE Healthcare, Cat. 80-6501-23), a chemiluminescent detection system (ECL Plus; Amersham Biosciences Cat. RPN2132), Lipofectamine 2000 (Invitrogen, Cat. 11668-019), an siRNA Labeling Kit-Cy3 (Ambion by Life Technologies Cat. AM1632), Sp1 siRNA (Sta. Cruz Cat. sc-29488), siRNA β_1_ and β_2_ (FlexiTube siRNA QIAGEN: SI04284966, SI04249098, SI04173134, SI03149909, SI04273003, SI04138162, SI04274543, SI04284014), the Light Cycler-Fast Start DNAMaster SYBR Green I Kit (Roche, (Applied Biosystems, 4309159), and BCA protein assay reagent (Thermo Scientific, 23224 and 23223).

The following antibodies were used: anti-Na^+^, K^+^-ATPase α_1_ subunit (IF: Novus NB300-146), anti-α_2_ Na^+^, K^+^-ATPase (Thermo Scientific, PA5-25725), anti-Na^+^, K^+^-ATPase β_1_ subunit (IF: Bio Reagents Cat. No. MA3-93; WB: Novus 464.8), anti-Na^+^, K^+^-ATPase β_2_ subunit (WB: Transduction Laboratories Cat. No. BD610915, IF: Biorbit orb10952 and Creative Biolabs MOB-3916z), anti-Na^+^, K^+^-ATPase β_3_ subunit (Transduction Laboratories Cat. No. BD610992), anti-Ezrin (Sigma E1281), anti-β-catenin (Invitrogen 13-8400), anti-N-cadherin (IF: ZYMED Cat. No. 333900), anti-CD147 (Bioscience No. Cat. 555961), Alexa 488- or 594-conjugated donkey anti-mouse or anti-rabbit IgG (Invitrogen, A11094, A21207, A21203, and A21202), TO-PRO (Invitrogen, T3605), Hoechst (Invitrogen H21491), peroxidase-conjugated anti-mouse and anti-rabbit antibodies (Zymed California, Cat. 62-6520 and 62-6120), and FITC-phalloidin (Sigma Chemical).

### Cell culture

The ARPE-19 cell line (ATCC CRL-2302) was originally obtained from a spontaneously transformed human RPE primary culture. We only used cells from the 5th to the 20th passages, when the cultures grew rapidly and formed cobblestone monolayers. Cells were maintained in 6-cm-diameter culture dishes in Dulbecco's Modified Eagle's Medium (DMEM) supplemented with fetal bovine serum (FSB; 10%), penicillin (100 U/ml) and streptomycin (100 μg/ml). The cultures were incubated in an atmosphere of 95% air with 5% CO_2_ at 37°C. The cells were propagated on 6.5-mm or 24-mm-diameter Millicell Hanging Cell Culture Inserts (0.4 μm pore) (Transwell Corning Incorporated cat. 3450 and 3470) that had been previously coated with laminin (10 μg/ml) and were maintained in 1:1 Dulbecco's Modified Eagle's Medium and Ham's F-12 medium (DMEM:F-12) supplemented with FBS (10%) for 1 week. For the remaining time, they were supplemented with FBS (1%) and ITS until a polarized monolayer was formed (4–6 weeks). These culture conditions were defined as re-morphogenic conditions.

### Transepithelial resistance (TER)

The degree of tight junction (TJ) sealing to ionic solutes was assessed by measuring the TER of cells grown for 6 weeks on transwell-permeable supports using an epithelial volt-ohmmeter (EVOM; World Precision Instruments Inc., Sarasota, FL). All measurements were performed at room temperature. Final values were obtained by subtracting the resistance of the bathing solution and the empty insert, and the results are expressed as the mean ± SE in ohms times centimeters squared (Ω•cm^2^).

### Immunofluorescence (IF), immunocytochemistry and confocal microscopy

IF assay were performed using monolayers grown on 6.5-mm transwells covered with laminin that were washed with PBS (phosphate-buffered saline) and fixed with ice-cold methanol for 10 min. The cells were then soaked in blocking solution (PBS containing 3% BSA) for 1 h at 37°C or overnight at 4°C. Then, cells were incubated with the primary antibodies for 60 min at 37°C, washed quickly 7 times with PBS, and then incubated with the secondary antibodies for 45 min at 37°C. All antibodies were diluted in blocking solution, and the following secondary antibodies were used: Alexa 488- or 594-conjugated donkey anti-mouse or anti-rabbit IgG. Nuclei were counterstained with TO-PRO dye and then washed twice. To detect filamentous actin, the cells were fixed in paraformaldehyde and labeled with FITC-phalloidin.

Human eye preparations were obtained from the ophthalmic pathology service at the “Dr. Luis Sánchez Bulnes” APEC hospital in Mexico City. Paraffin-embedded eyes without lesions in their fundus were selected from the service compendium, and 5-μm sections were mounted on slides. For Na^+^, K^+^-ATPase assessment, β_2_ subunit-stained sections were deparaffinized and rehydrated using a series of incubations with xylene (2–5 min and 1:1 xylene/ethanol) and ethanol (2 min each in 100, 95, 80, and 70% ethanol), followed by three washes in PBS or water. The samples were bleached via incubation in 0.25% KMnO_4_ and PBS-Ca^2+^ for 30 min. After three washes with PBS-Ca^2+^, the samples were incubated in 1% oxalic acid and washed again. Antigen retrieval was accomplished via incubation in 0.05% trypsin for 10–30 min at 37°C. Sections were permeabilized using 0.25% Triton X-100 and 1% FBS for 30 min and then blocked with 0.25% Triton X-100 and 10% FBS for 90–120 min. The samples were incubated with primary antibodies (against CD147 and the α2 and β_2_ subunits of Na^+^, K^+^-ATPase) in permeabilization solution overnight at 4°C. The next day, the samples were washed three times and incubated with a secondary antibody for 1 h at 37°C. The samples were washed twice, and the nuclei were counterstained with DAPI.

Confocal microscopy was performed using a Leica laser-scanning confocal microscope (Leica TCS SP2 or TCS SP8). Data acquisition and analysis were performed with the LCS Leica software and ImageJ® software from the National Institutes of Health (Bethesda, MD), respectively. The relative fluorescence intensity was quantified using ImageJ 1.43u software.

### Western blot (WB) analysis

All extraction steps were performed at 4°C. To detect the protein levels of the Na^+^, K^+^-ATPase subunits, monolayers grown on 24-mm transwells covered with laminin were lysed in a buffer containing 40 mM Tris (pH 7.6), 150 mM NaCl, 2 mM EDTA, 10% glycerol, 1% Triton X-100, 0.5% Na^+^ deoxycholate, 0.2% SDS, and protease inhibitors (Complete, Mini). The extract was sonicated for 30 s and centrifuged at 15,000 × g in a microfuge for 15 min. The supernatant was recovered, and the protein content was measured using BCA protein assay reagent following the manufacturer's instructions. Thirty micrograms of protein from each condition were separated via 10% SDS-PAGE and immunoblotted with the indicated primary antibodies, followed by species-appropriate peroxidase-conjugated secondary antibodies, which were imaged using a chemiluminescence detection system. The immunoblots were quantified via densitometry using ImageJ 1.43u software.

### Steady state surface biotinylation assay

ARPE-19 cells were maintained for 4 weeks in a culture on polyester transwell inserts as described above. Cell monolayers were biotinylated with 1 mg/ml of EZ-Link Sulfo-NHS-SS-Biotin (Thermo scientific, 21331). After quenching the biotinylation reaction, the cells were washed and then lysed, and the membranes were solubilized by incubating them with 200 μl of PBS (pH 8.0) with 1% Triton X-100 and protease inhibitors. Cell lysates were clarified via centrifugation (15,000 × g for 10 min). Samples containing 50 μl of supernatant mixed with SDS-containing sample buffer were loaded into SDS-PAGE gels to determine the total ARPE-19 protein in the supernatant (input). To isolate biotinylated proteins, the rest of each supernatant was incubated with 100 μl of streptavidin-agarose beads (Gibco, 5942SA) in a total volume of 150 μl of lysis buffer overnight at 4°C with continuous rotation. The bead-adherent complexes were washed 6 times [PBS (pH 8.0), 1% Triton X-100 and 150 mM NaCl]. Next, the proteins were eluted from the beads via incubation in SDS-PAGE sample buffer for 5 min at 80°C, separated in SDS-PAGE gels and analyzed via WB analysis using primary antibodies against human Na^+^, K^+^-ATPase β subunits and human N-cadherin.

### Transfection of siRNA

ARPE-19 cells were cultured for 4 weeks and incubated for 48 h with a mixture of Lipofectamine 2000 and 278 ng/μl of siRNA for the β_1_ subunit, 700 ng/μl of siRNA for the β_2_ subunit or 75 pmoles/μl of siRNA for Sp1, as indicated by the manufacturer. Thereafter, the transfection medium was removed, and the cells were processed for IF or WB analysis to estimate the silencing efficiency. siRNA for the Na^+^, K^+^-ATPase β_2_ subunit and for Sp1 were pre-labeled with Cy3 using the siRNA Labeling Kit-Cy3 according to the manufacturer's protocol.

### Relative mRNA quantification via qPCR

Real-time PCR was performed with a Light Cycler 2.0 system (Roche) using the Light Cycler-Fast Start DNA Master SYBR Green I Kit (Applied Biosystems). We used the following sets of primers: β_2_ subunit forward: GAGCTTCGTTCCACAGCTTC and reverse: CCCACCAAACCGTCTAGA AA; β_1_ subunit forward: AGGCGTACGGTGAGAACATT and reverse: GGGAAAGATTTGTGCTTG TGA; β_3_ subunit forward: TCGAGTACTCCCCGTAACGA and reverse: AGGCTCTGGTTGAGGGAC TT; α_1_ forward: GAAGCAAGACGTCCTGGAAT and reverse: TTTCAGTCTTTCCGGGTGTT; α_2_ forward: CTACCCTGTTGCTTTGGCTTTC and reverse: TGAGGGACCTTAGCGGGAGA; and GAPDH forward: ACGGCACAGTCAAGGCTGAG and reverse: CAGCATCACCCCATTTGATGTTGG. PCRs were performed using 45 cycles that included the following steps: 30 s of denaturation at 95°C, a 30-s annealing phase at 60°C, and a 30-s template-dependent elongation phase at 72°C. The amplification of each DNA template was performed in at least three experiments with two technical replicates in the same PCR run. The differential gene expression of the investigated genes was calculated as the ratio normalized to the expression of the GAPDH gene. The data were analyzed using the equation described by Livak (Livak and Schmittgen, 2001; amount of target = 2^−ΔΔCT^).

### Statistical analysis

GraphPad Prism version 4.00 software was used for all statistical analyses. The data are presented as the mean ± SEM. Statistical significance was determined using a one-tailed, non-parametric *t*-test. *P* ≤ 0.05 were considered significant.

## Results

### The β_2_-subunit of Na^+^, K^+^-ATPase is expressed at the apical domain of the RPE in the eye

**Figure 1:**
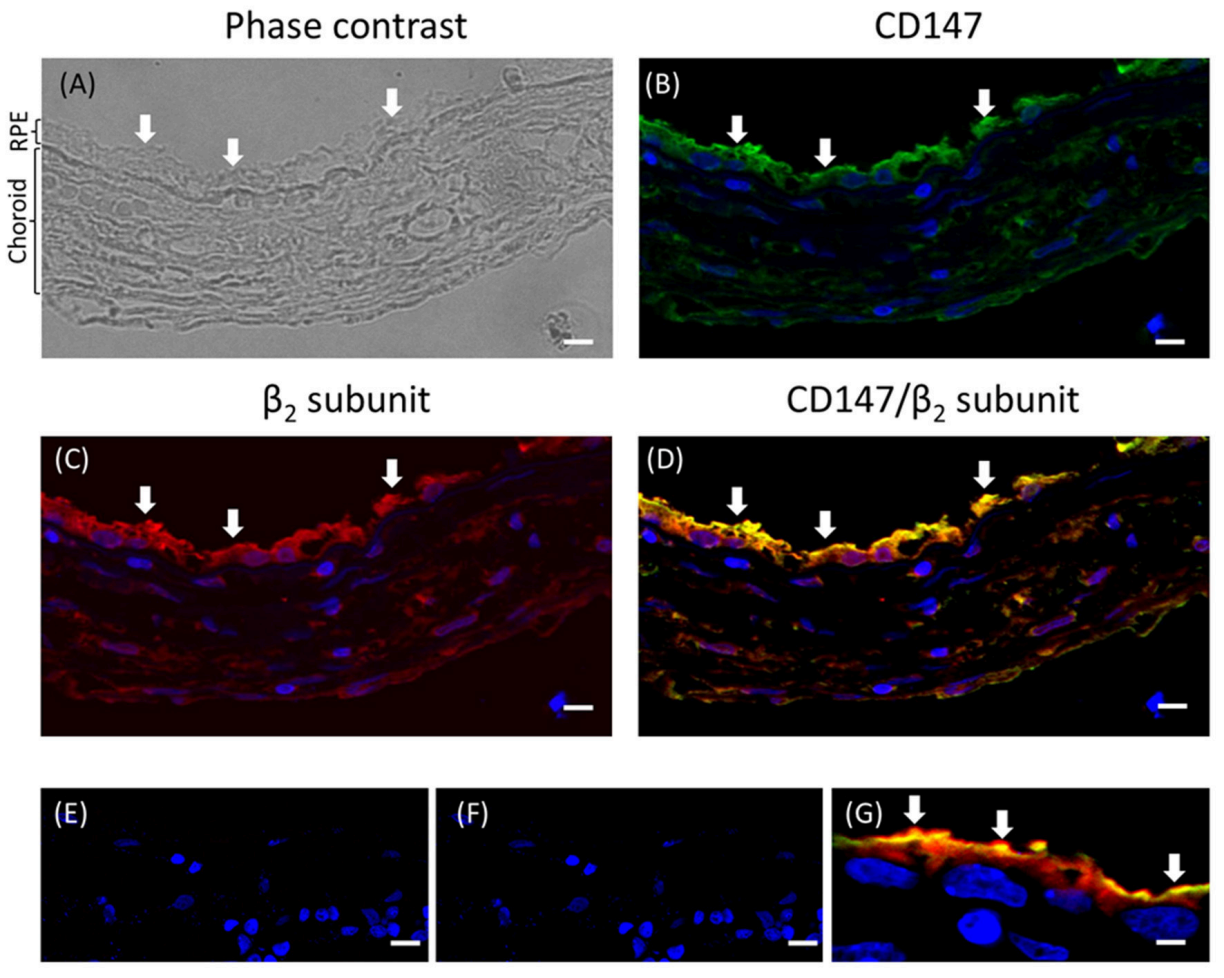
**Immunofluorescence of adult human eye ***in situ***. (A)** A phase-contrast image of the human eye section studied. The RPE layer and the choroid are indicated. The retina is already detached in the paraffin block. This section was co-stained for CD147 **(B)** and the β_2_ subunit of the Na^+^, K^+^-ATPase **(C)** using Alexa 488- and Alexa 594-conjugated donkey anti-rabbit and anti-mouse IgG secondary antibodies, respectively. The merged image showing co-localization at the apical domain is in **(D)**. Panels **(E,F)** show similar sections treated only with the fluorescent secondary antibody (anti-rabbit and anti-mouse IgG, respectively) as negative controls. Panel **(G)** shows a higher-magnification image from a different field of the same preparation. All the preparations were counterstained with DAPI (blue). Arrows indicate the apical domain of RPE cells. Scale bars are 40 μm in **(A–F)** and 10 μm in **(G)**.

To test the hypothesis that the apical targeting of Na^+^, K^+^-ATPase in RPE cells involves the expression of the β_2_ subunit, we first analyzed the expression of the β_2_ isoform at the apical membrane of the RPE in the eye. As shown in sections from human eye (Figure 1), co-localization at the apical domain was observed using anti-β_2_ antibody and anti-CD147 antibody (basigin or cluster of differentiation 147, the accessory subunit of monocarboxylate transporters; 35). Thus, our data suggest that the apical Na^+^, K^+^-ATPase expressed in human RPE includes the β_2_ isoform.

### ARPE-19 cells are suitable for studying the mechanism underlying the polarity of Na^+^, K^+^-ATPase in the RPE

**Figure 2:**
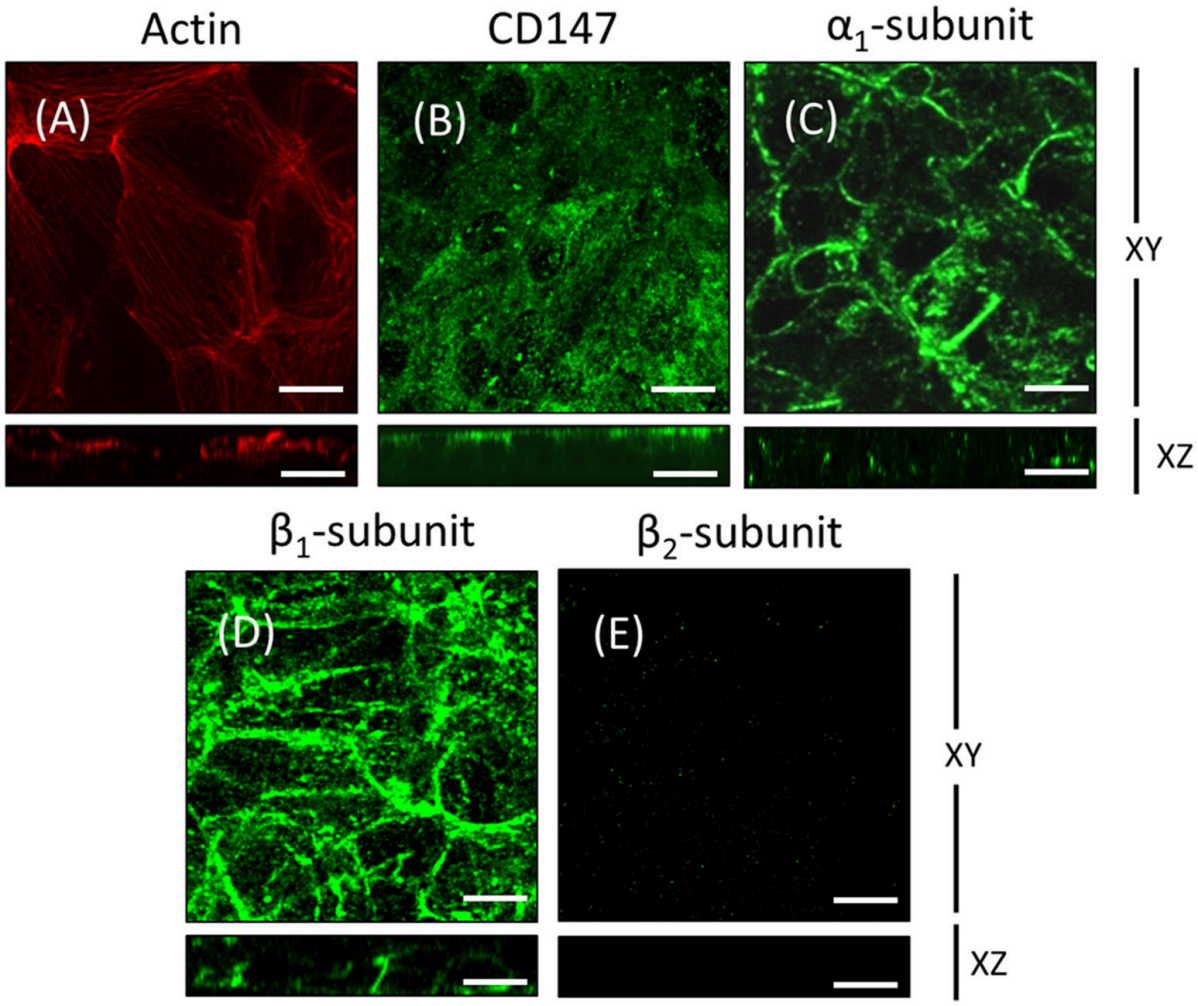
**ARPE-19 cells cultured on transwell inserts for 4 weeks are not completely polarized**. ARPE-19 cells were cultured up to 4 weeks on transwell inserts covered with laminin. The immunofluorescence image in **(A)** shows actin localization using rhodamine phalloidin. The cells are flat with stress fibers and very little circumferential actin microfilament bundles. The expression of CD147, a RPE marker, was detected using a specific antibody in the apical membrane domain **(B)**. The expression of Na^+^, K^+^-ATPase using anti-α_1_ **(C)** and anti-β_1_ antibodies **(D)** was observed mostly at the basolateral membrane. Immunofluorescence detection with anti-human β_2_ antibody revealed a very weak signal **(E)**. Scale bar: 10 μm.

**Figure 3:**
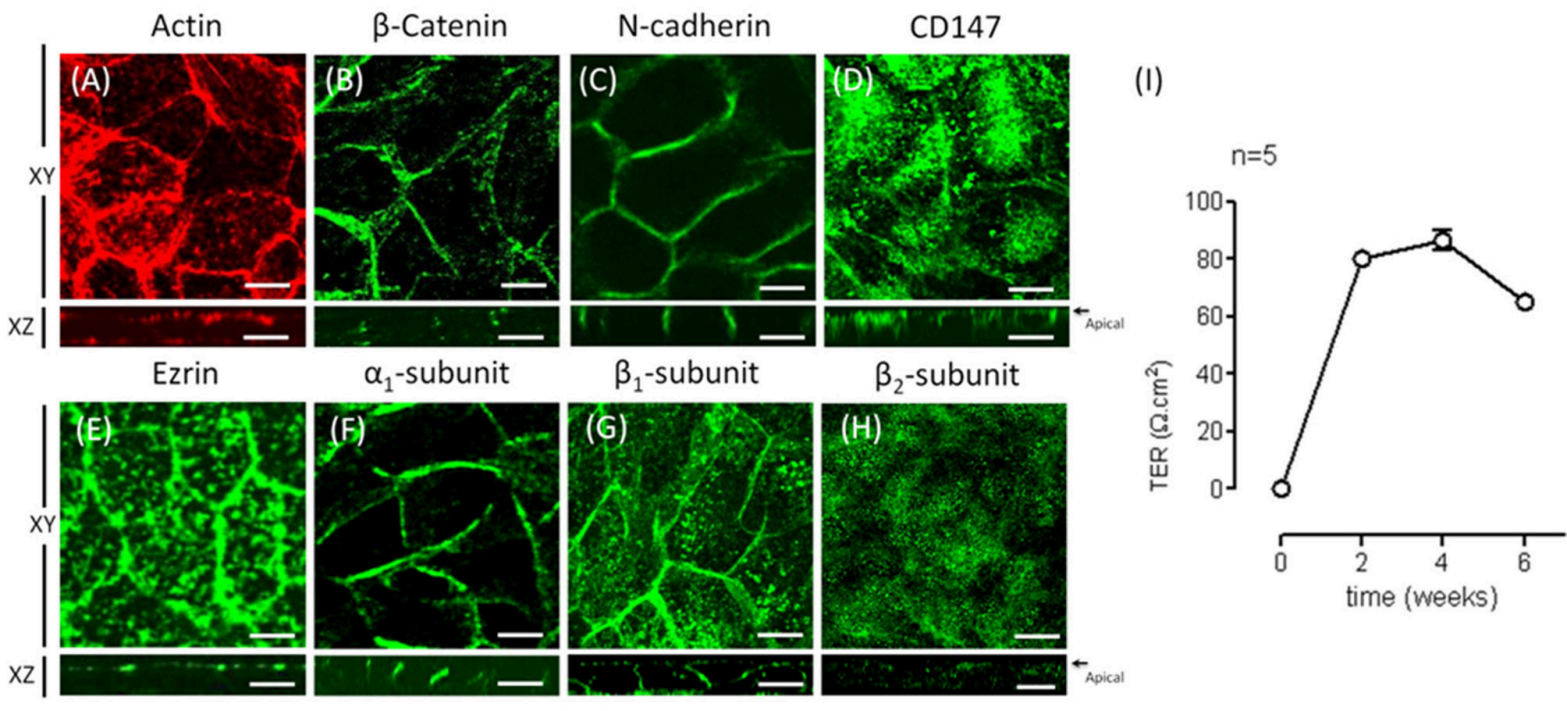
**Re-morphogenesis of ARPE-19 cells treated with ITS. (A)** The photomicrographs show ARPE-19 cells cultured for 4 weeks with ITS and stained for actin with Texas Red-X phalloidin. Note the tight intercellular contact, epithelial-like cellular shape, and predominantly peripheral cortical actin staining. Confocal immunofluorescence images of the same culture conditions showing β-catenin **(B)** and N-cadherin **(C)** at the lateral membrane, CD147 **(D)** mostly in the apical domain and ezrin **(E)** in the apical domain. **(F)** The immunofluorescence image shows immunostaining of the α_1_ subunit of Na^+^, K^+^-ATPase at the lateral membrane and more precisely, as observed in the XZ image, at the cell-cell contacts. **(G)** The β_1_ subunit is observed at the cell border and at the apical domain; the XZ image confirms both basolateral and apical staining. **(H)** The β_2_ subunit is observed mainly at the apical domain; the XZ image confirms apical staining. Scale bar: 10 μm. **(I)** Quantitative analysis of the transepithelial resistance (TER) of ARPE-19 cells during re-morphogenesis is depicted. Note a stable TER of ~80 Ω cm^2^ between the second and fourth week in culture.

To further test our hypothesis, we chose human ARPE-19 cells as a model. ARPE-19 cells are fibroblast-like when cultured on inserts and go through a process of re-morphogenesis that lasts 6–8 weeks (Dunn et al., 1996). This period probably reflects the time required to up-regulate the expression of genes associated with differentiated RPE cells and is needed to develop the molecular machinery involved in membrane protein localization in RPE cells. First, we analyzed the polarized expression of Na^+^, K^+^-ATPase in ARPE-19 cells. In ARPE-19 cells cultured up to 4 weeks on transwell inserts covered with laminin, immunofluorescent staining of actin using rhodamine phalloidin showed flat cells with stress fibers and very few circumferential actin microfilament bundles (Figure 2A). The expression of CD147, was detected in the apical membrane domain using a specific antibody (Figure 2B). The expression of Na^+^, K^+^-ATPase using anti-α_1_ and β_1_ antibodies was mostly observed in the basolateral membrane (Figures 2C,D). Furthermore, using anti-human β_2_ antibody, a very weak signal was detected (Figure 2E). Under these culture conditions, we were unable to study the mechanism underlying the apical polarization of the Na^+^ pump. Thus, we decided to add ITS, which has been reported to epithelialize ARPE-19 cells (Luo et al., 2006). We then examined whether this supplement resulted in the apical localization of Na^+^, K^+^-ATPase. As shown in Figure 3, after 4 weeks of culturing, cells cultivated in the presence of ITS were epithelial-like in shape, with a circumferential actin microfilament bundle and occasional stress fibers (Figure 3A). As expected for RPE cells, the expression of molecular markers such as β-catenin and N-cadherin was observed in the lateral membrane (Figures 3B,C), and CD147 was observed at the apical and basolateral membrane (Figure 3D). Ezrin, a membrane-organizing phosphoprotein that tethers actin microfilaments to cell membrane proteins, is an apical polarization marker in the RPE (Kivelä et al., 2000). As shown in Figure 3E, after 4 weeks of culturing in the presence of ITS, ezrin was localized at the apical membrane in a pattern suggesting the formation of microvilli. The expression of Na^+^, K^+^-ATPase assessed using anti-α_1_ and anti-β_1_ antibodies (Figures 3F,G) was mainly observed in the basolateral domain, although β_1_ expression was also observed in the apical domain. An apical pattern was observed using anti-β_2_ antibody (Figure 3H). TJ formation was evaluated based on measurement of the TER of the monolayers. As depicted in Figure 3I, the TER was stabilized at 4 weeks, with an average value of 80 Ω•cm^2^, which is a characteristic value reported in these cells (Dunn et al., 1996; Luo et al., 2006). Hence, we considered that under these conditions (designated as re-morphogenic conditions and detailed in the Methods), it would be feasible to perform experiments addressing the intriguing issue of the “reversed” apical polarization of Na^+^, K^+^-ATPase in RPE cells.

### Expression of the α_2_ and β_2_ isoforms is up-regulated during re-morphogenesis

**Figure 4:**
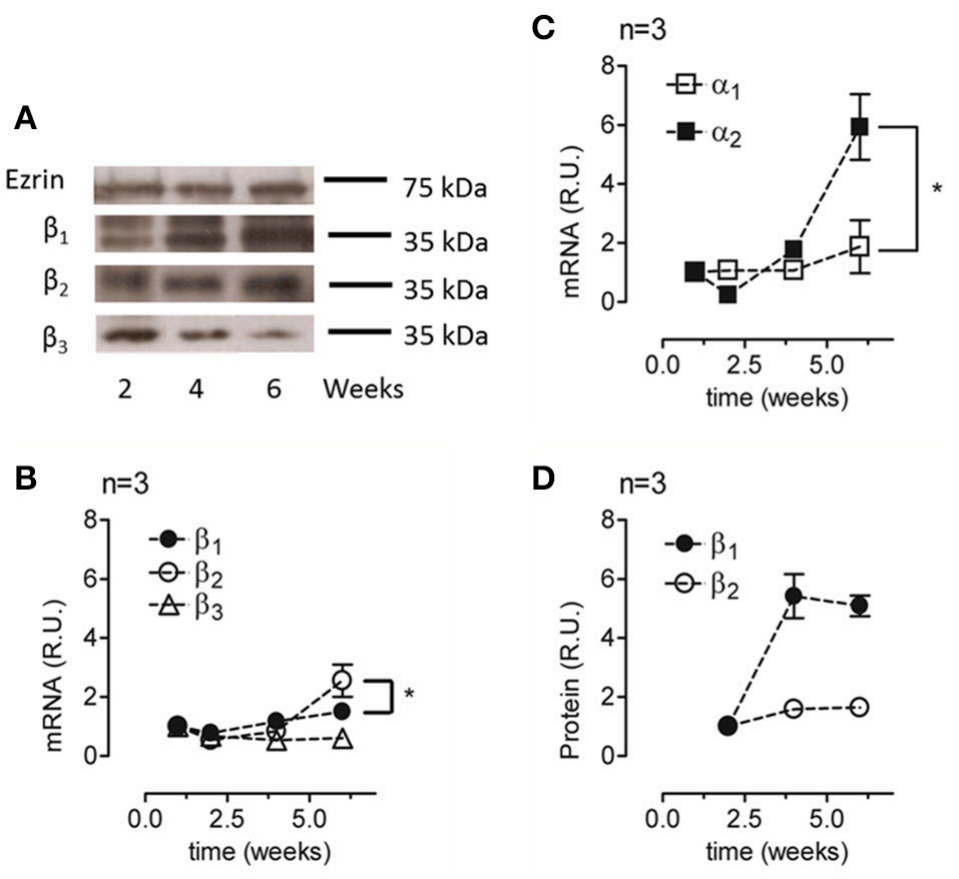
**Analyses of the relative amounts of α and β isoforms in ARPE-19 cells during re-morphogenesis. (A)** Western blot analysis of the lysates of ARPE-19 cells cultured for the indicated time under conditions established for re-morphogenesis. The upper part of the blot was probed with an antibody against ezrin (as a loading control), whereas the lower part was probed with antibodies against the β_1_, β_2_, and β_3_ subunits. Western blot analyses of all samples were conducted on the same day. A non-relevant lane between 2 and 4 weeks in the ezrin blot was eliminated; therefore, it appears to be discontinued. The blots represent three different experiments. Total mRNA was extracted at weeks 1, 2, 4, and 6 of culture and analyzed via qPCR. The relative mRNA levels of the β_1_, β_2_, and β_3_ subunits **(B)** and of the α_1_ and α_2_ subunits **(C)** are illustrated. The amount of mRNA was normalized to that detected in the first week. **(D)** Quantification of β_1_ and β_2_ subunit expression via densitometry in three independent experiments normalized to the loading control. Densitometry results were also corrected for the film light level whenever the background level was different. Error bars represent the mean ± SEM of three independent experiments. Significant changes are indicated by an asterisk (*P* < 0.05, non-parametric *t*-test).

To characterize the expression of Na^+^, K^+^-ATPase under re-morphogenic conditions, we analyzed the mRNA and protein expression levels of various isoforms of the α and β subunits. The applied antibodies were carefully chosen to ensure that they were specific for the designated isoform. As shown in Figure 4A, ARPE-19 cells cultured in the presence of ITS expressed the three β subunits (β_1_, β_2_, β_3_). Remarkably, these findings are the first evidence of β_3_ isoform expression in ARPE-19 cells. We then evaluated changes in the amount of mRNA for the α and β isoforms over time via qPCR. As shown in Figures 4B,C, mRNAs corresponding to all five studied isoforms (α_1–2_, β_1–3_) were expressed in ARPE-19 cells, and their relative amounts increased during re-morphogenesis. As shown in Figure 4B, although the relative amounts of β_1_ and β_2_ mRNA increased up to the sixth week, the mRNA levels of β_2_ increased to a significantly higher value than those of β_1_. However, the amount of β_3_ mRNA remained relatively constant during this time period (Figure 4B). Therefore, we did not study this isoform further. Nevertheless, while the amount of α_1_ mRNA was doubled, the expression of α_2_ increased 6-fold (Figure 4C). When analyzing the amounts of total protein of the β_1_ and β_2_ isoforms, we observed an increase over time, reaching maximal expression at 6 weeks. Altogether, Figure 4 shows that during re-morphogenesis, the expression of the β_2_ subunit was up-regulated, resulting in increasing amounts of both mRNA and protein. This suggests transcriptional level regulation.

### The transcription factor Sp1 is involved in regulating the expression of the β_2_ subunit in ARPE-19 cells

**Figure 5:**
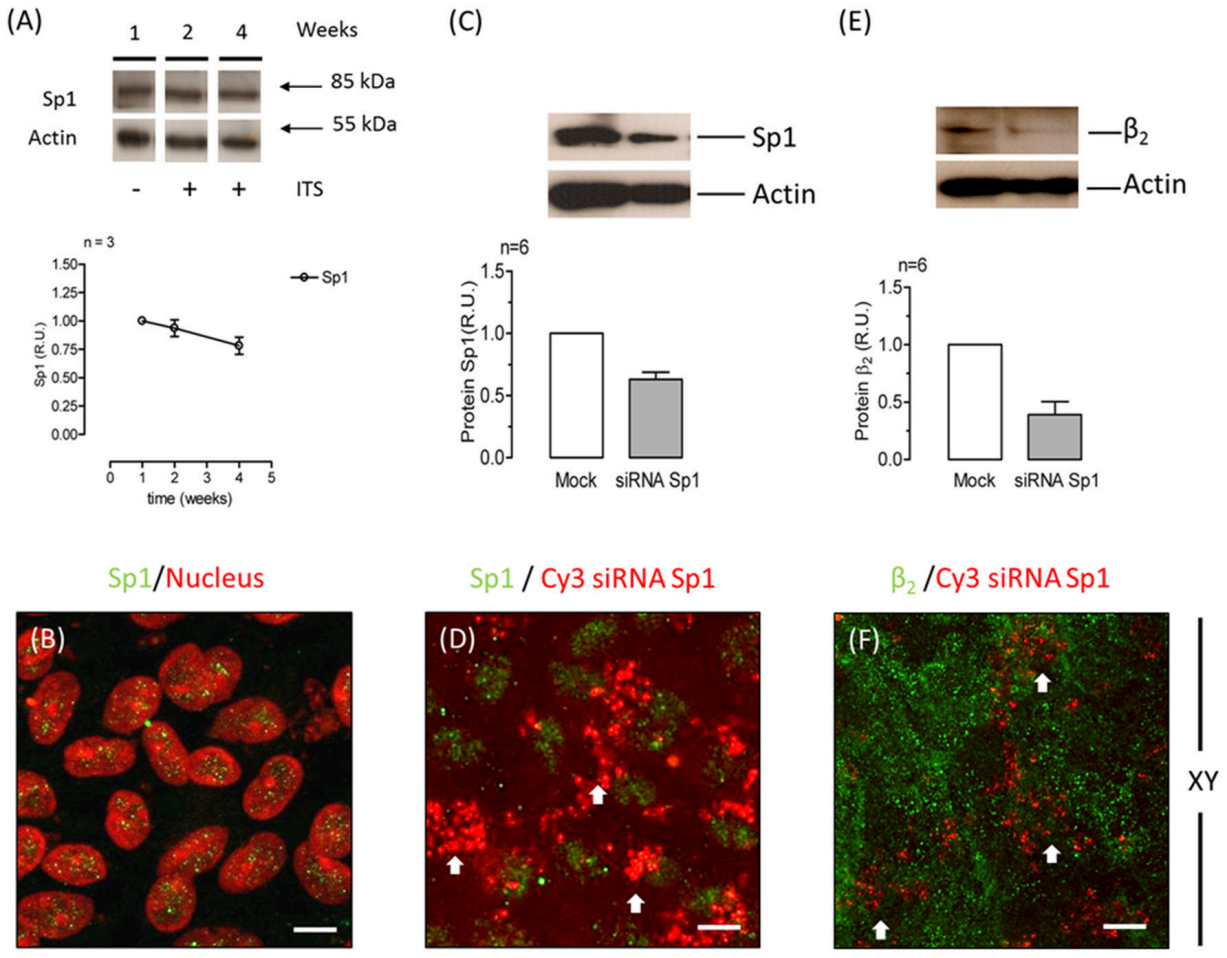
**The transcription factor Sp1 is involved in β2 subunit regulation in ARPE-19 cells. (A)** Western blot analysis showing the expression of Sp1 over 4 weeks of culturing (upper panel). Actin was used as a loading control in all Western blots shown in this figure. Quantitative analysis of Sp1 normalized to the loading control is illustrated in the lower panel. **(B)** Immunofluorescent staining of Sp1 in ARPE-19 cells is shown in green. Counterstaining of nuclei is shown in red. Merged image shows the nuclear localization of Sp1 in ARPE-19 cells cultured for 4 weeks in the presence of ITS. Scale bar: 20 μm. **(C)** Representative Western blot and quantitative analyses of six independent experiments conducted on ARPE-19 cells treated with Sp1 siRNA are shown. Control cells were transfected without siRNA (Mock). Error bars represent the mean ± SEM. **(D)** Immunofluorescence image of ARPE-19 cells incubated with Cy3-siRNAs to silence Sp1 (red) and immunostained for Sp1 expression (green). White arrows indicate siRNA-transfected cells (identified by red fluorescence) that did not express Sp1 in the nucleus. Scale bar: 20 μm. **(E)** Representative Western blot and quantitative results of the immunodetection of the β_2_ subunit in six independent experiments of Sp1 knockdown in cells (60%). **(F)** Immunofluorescence image of Sp1-silenced ARPE-19 cells stained for β_2_ subunit expression.

During re-morphogenesis, expression of the α_2_ and β_2_ isoforms is up-regulated, increasing both their mRNA and protein levels. The transcription factor specificity protein 1 (Sp1) binds GC-rich motifs and regulates gene expression through protein–protein interactions (Shull et al., 1989; Samson and Wong, 2002). Based on previous works by Kawakami et al. (1990, 1992) and Avila et al. (1998) that reported that Sp1 enhances the promoter activity of the β_2_ subunit in rat neuroblastoma, in rat embryo cell lines and in human lymphocytes, we suspected that Sp1 could be at least one of the factors regulating this process. Therefore, we explored whether Sp1 was involved in the up-regulation of the β_2_ subunit during the re-morphogenesis of ARPE-19 cells. As shown in Figure 5A, the relative amount of Sp1, as estimated via WB analysis, was slightly changed during re-morphogenesis. IF images in Figure 5B show that Sp1 was expressed in the nuclei of ARPE-19 cells cultured for 4 weeks in the presence of ITS. The silencing of Sp1 by siRNAs specific for human Sp1 in ARPE-19 cells (cultured for 4 weeks) reduced the total protein level, as estimated from the WBs, by ~40% (Figure 5C). This partial silencing corresponds to the IF image of the silenced cells in Figure 5D. The arrowheads indicate cells in which the expression of Sp1 (in green) was not observed, although these were still surrounded by cells that did express Sp1 in their nuclei. We anticipated that if Sp1 was involved in β_2_ transcription, Sp1 silencing would also reduce the amount of the β_2_ subunit in ARPE-19 cells. As shown in Figure 5E, the total amount of β_2_ estimated via WB analysis was reduced by ~50% in Sp1-silenced cells. Correspondingly, the IF image of β_2_ subunit expression (Figure 5F) shows zones in the Sp1-silenced monolayer with low fluorescence signal (indicated by arrowheads). These data suggest that the transcription factor Sp1 is probably involved in regulating the expression of the β_2_ subunit in ARPE-19 cells.

### Apical expression of Na^+^, K^+^-ATPase in ARPE-19 cells during re-morphogenesis is correlated with the expression of the α_2_ and β_2_ isoforms

**Figure 6:**
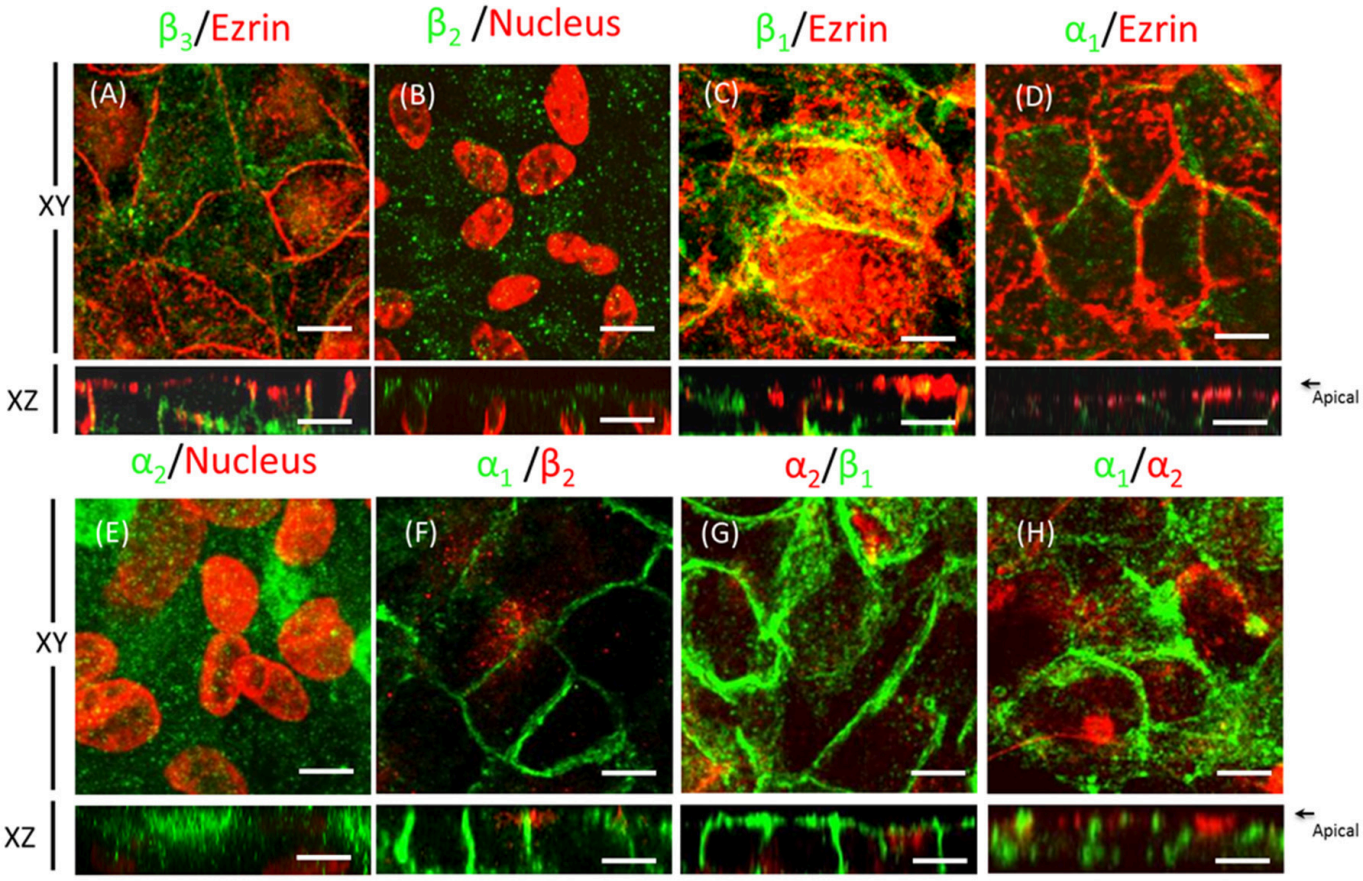
**Domain-specific distribution of the Na^**+**^, K^**+**^-ATPase isoforms in ARPE-19 cells**. Immunofluorescence assays of ARPE-19 cells cultured for 4 weeks with ITS. **(A–E)** Fluorescence images of immunostaining with isoform-specific antibodies against the α and β subunits of Na^+^, K^+^-ATPase; co-staining with anti-ezrin as an apical marker **(A,C,D)** or counterstaining with propidium iodide for the detection of nuclei **(B,E)**. β_3_ **(A)** and α_1_ **(D)** subunit expression was detected mainly in the lateral domain. The distribution pattern of the β_2_ and α_2_ subunits **(B,E)** suggests an apical localization. The distribution pattern of the β_1_ subunit **(C)** in both the lateral and apical domains suggests non-polarized expression. Minor co-localization with ezrin at the apical domain is observed. The α_1_/β_2_ **(F)**, α_2_/β_1_ **(G)** and α_1_/ α_2_ subunits **(H)** do not co-localize. Scale bar: 10 μm.

**Figure 7:**
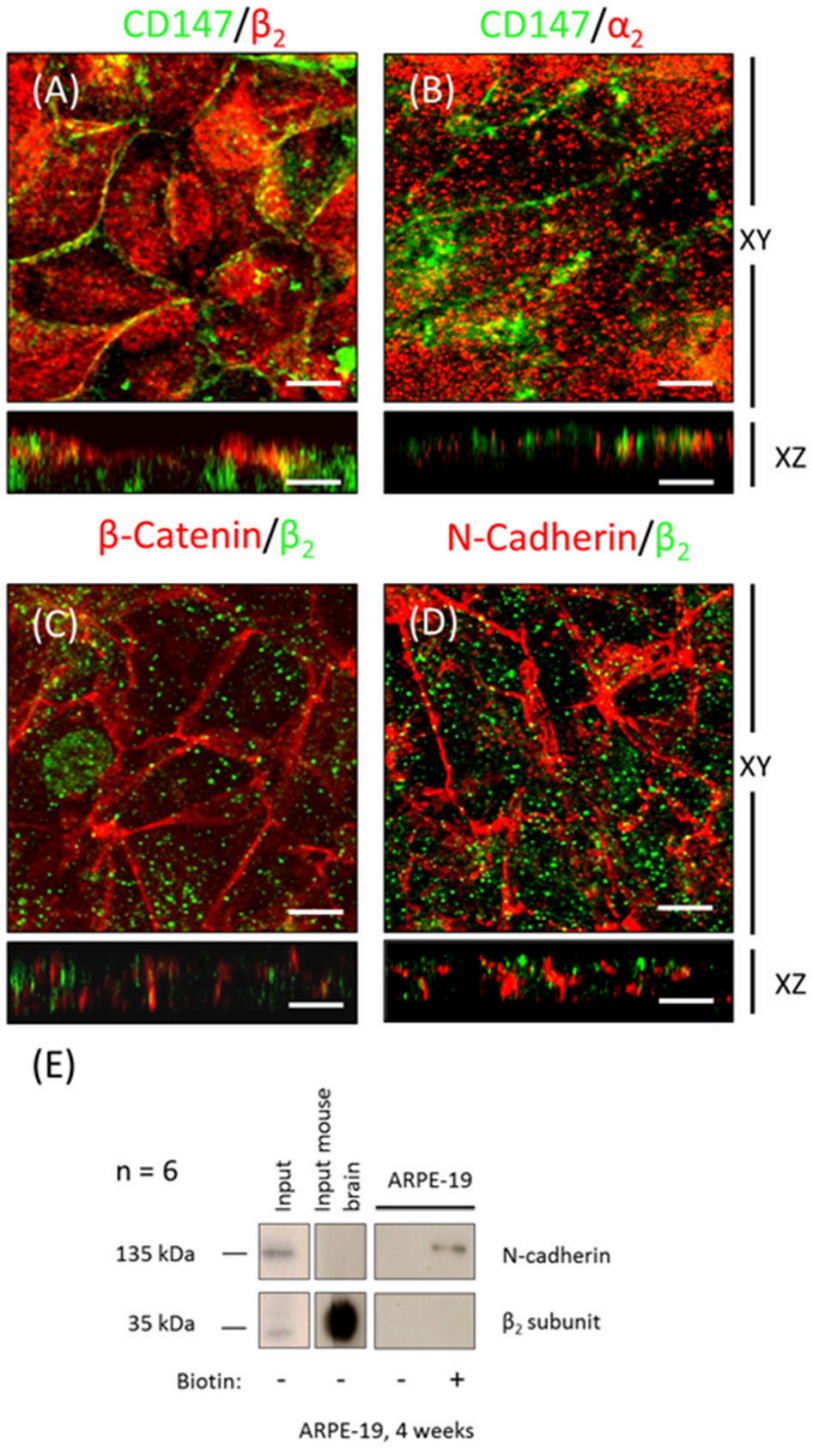
**The β_**2**_ subunit in ARPE-19 cells does not stabilizes at the apical plasma membrane domain**. Confocal images of the β_2_ and α_2_ subunits (in red) were analyzed for co-localization with CD147 (green), an apical marker **(A,B)**. Co-localization of the basolateral markers β-catenin and N-cadherin (in red) with the β_2_ subunits (in green) are shown in **(C,D)**. Although an apparently apical pattern is observed for the Na^+^, K^+^-ATPase subunits β_2_ and α_2_, they do not co-localize with either apical or lateral markers. Scale bar: 10 μm. Representative Western blot of six independent experiments conducted in ARPE-19 cells for steady state surface labeling with biotin is presented in **(E)**. Notice the lack of a β_2_ subunit band in the biotin labeled lane.

**Figure 8:**
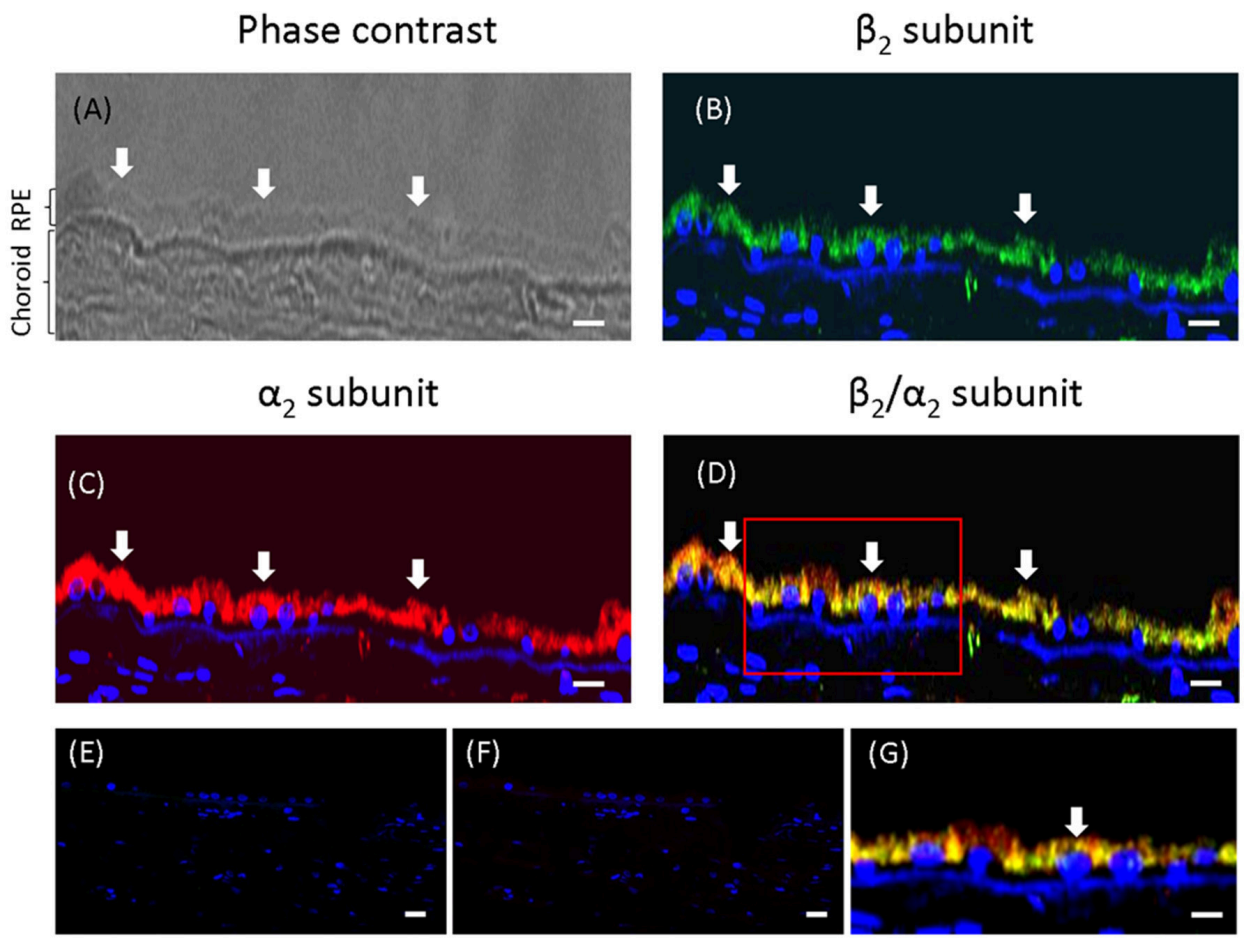
**Co-localization of the α_**2**_ and β_**2**_ subunits in adult human eye. (A)** A phase-contrast image of the human eye section studied. The RPE layer and the choroid are indicated. This section was co-stained for the β_2_ **(B)** and α_2_ subunits **(C)** of Na^+^, K^+^-ATPase using Alexa 488- and Alexa 594-conjugated donkey anti-rabbit and anti-mouse IgG secondary antibodies, respectively. The merged image showing co-localization at the apical domain is in **(D)**. Panels **(E,F)** show images of similar sections treated only with the secondary fluorescent antibody (anti-rabbit and anti-mouse, IgG, respectively) as a negative control. Panel **(G)** shows the field indicated by the square in **(D)** at higher magnification. All the preparations were counterstained with DAPI (blue). Arrows indicate the apical domain of RPE cells. Scale bars are 25 μm in **(A–D)**, 40 μm in **(E,F)** and 30 μm in **(G)**.

The WB and qPCR results in Figure 4 show that ARPE-19 cells expressed all three β isoforms and at least two α isoforms. We therefore proceeded to analyze the polarized distribution of the different α and β isoforms in these cells. As observed from the confocal IF analysis (Figure 6) and in contrast to the non-polarized distribution of the β_1_ isoform shown in Figure 3G, β_3_ was mostly localized at the basolateral membrane and did not co-localize with ezrin (Figure 6A). Therefore, we did not study the role of this isoform in the apical localization of the pump in RPE. The β_2_ subunit was distributed in a typical dotted pattern that suggested an apical localization (Figure 6B). The β_1_ subunit was distributed in both the basolateral and apical domains (Figure 6C). Nonetheless, the apparent apical pattern was not homogenous, presenting a mosaicism that has been previously reported for RPE cells (Burke, 2008). Because the β subunit must associate with the α subunit to reach the plasma membrane, we analyzed the α isoform accompanying the β_2_ subunit in ARPE-19 cells using an IF assay. Figure 6D shows that α_1_ was present at the basolateral membrane and was clearly excluded from the apical domain marked by ezrin. However, α_2_ was distributed in an apical pattern and was apparently excluded from cell-cell contacts (Figure 6E), very similar to the β_2_ pattern. We also observed a lack of co-localization between α_1_ and β_2_ (Figure 6F), α_2_ and β_1_ (Figure 6G) and α_1_ and α_2_ isoforms (Figure 6H). Thus, the apical pump in RPE cells is most likely an α_2_/β_2_ complex. Although the IF distribution pattern of the β_2_ subunit in polarized ARPE-19 cells suggests an apical localization, we had to confirm that the pump assembled by the β_2_ isoform was actually delivered to the apical membrane domain of ARPE-19 cells. Therefore, we examined the co-localization of the β_2_ and α_2_ subunits with the apical marker CD147 (Figures 7A,B) and with the basolateral markers N-cadherin and β-catenin (Figures 7C,D). Images obtained via confocal microscopy (Figure 7) show that the β_2_ subunit did not co-localize with markers of the basolateral or apical domains. However, a lack of co-localization demonstrated by IF was not sufficient to conclude that β_2_ does not reside at the apical membrane. Thus, we proceeded to perform a steady-state surface biotinylation assay. ARPE-19 cells were cultured for 4 weeks with ITS on inserts. Biotin was added to both the apical and basolateral sides of the monolayer. As shown in Figure 7E, these cells expressed both N-cadherin and the β_2_ subunit, as detected in the total cell lysate (input). N-cadherin was labeled with biotin as expected. Nevertheless, the β_2_ subunit was not detected in the biotin-labeled (streptavidin-precipitated) fraction in any of the 6 experiments performed. Therefore, our results indicate that apical pumps including β_2_ subunits probably did not accumulate in the apical membrane domain of ARPE-19 cells. To evaluate this possibility we used immunofluorescence assays to analyze whether α_2_ and β_2_ co-localize at the apical domain in sections of human eye. As shown in Figure 8, the α_2_ and β_2_ subunits co-localize in an apical domain. Considering that the β_2_ subunit is an adhesion molecule (Gloor et al., 1990), we speculated that it did not stabilize in the plasma membrane because it could not interact with a receptor protein at the apical “lumen” of the monolayer. Hence, the apical pumps observed via IF in ARPE-19 cells may result from apical recycling of β_2_ subunits accumulated in endosomes (AREs or CREs, 12). Interestingly, the β_1_ isoform was clearly detected when the biotin-labeled fraction of the ARPE-19 cells was blotted for the β_1_ subunit (data not shown), suggesting that the β_1_ isoform had a slower turnover in the plasma membrane and therefore was detectable in a steady-state analysis. However, these findings do not preclude the involvement of the β_2_ subunit in the apical polarization of Na^+^, K^+^-ATPase.

### Silencing the expression of the β_2_ isoform in mature ARPE-19 cells decreases the apical localization of Na^+^, K^+^-ATPase

**Figure 9:**
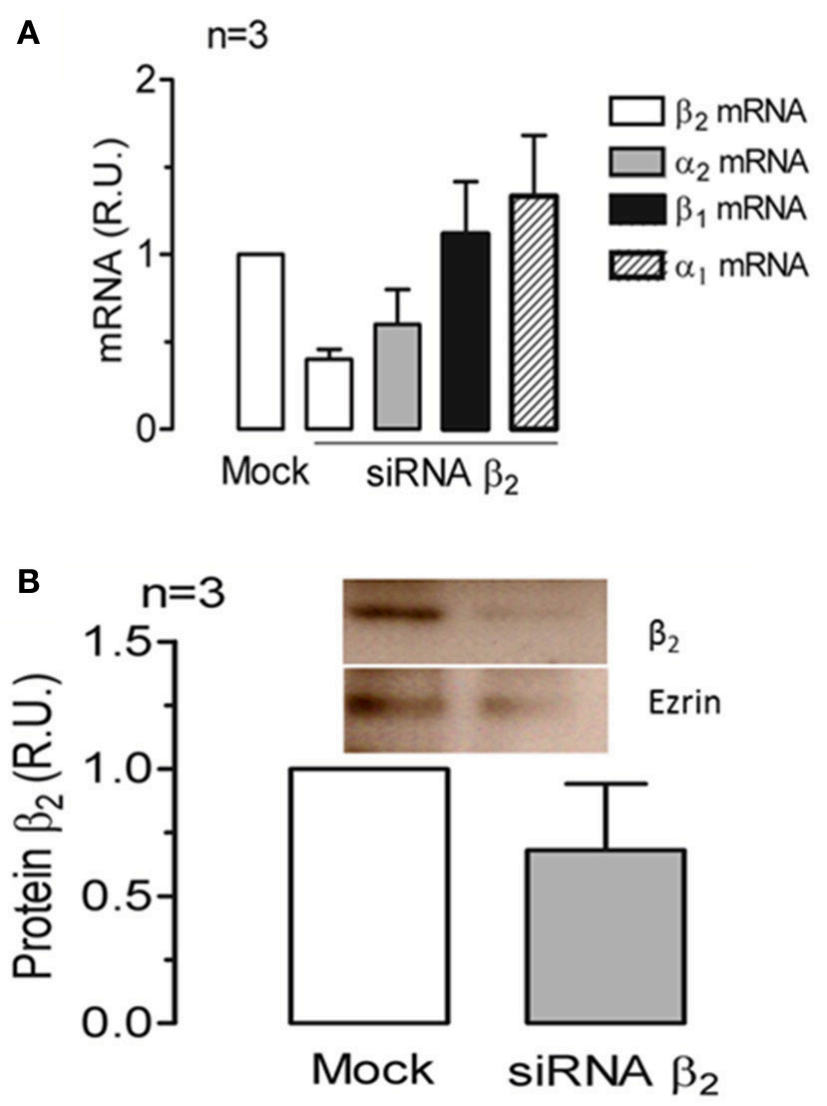
**siRNA silencing of the human Na^**+**^, K^**+**^-ATPase β_**2**_ isoform in mature ARPE-19 cells**. ARPE-19 cells cultured for 4 weeks with ITS were knocked down using siRNAs against the human β_2_ subunit. **(A)** mRNA levels of the α_1_, α_2_, β_1_, and β_2_ subunits were assessed in three independent experiments in ARPE-19 cells transfected with β_2_ siRNA or without any siRNA (Mock) via qPCR. The extent of β_2_ mRNA silencing was ~60%. **(B)** Western blot and quantitative results from silenced ARPE-19 cells treated as in **(A)** are presented. The amount of protein corresponding to the β_2_ subunit was normalized to the level detected in the mock-transfected cells. The extent of β_2_ protein silencing was ~40%.

**Figure 10:**
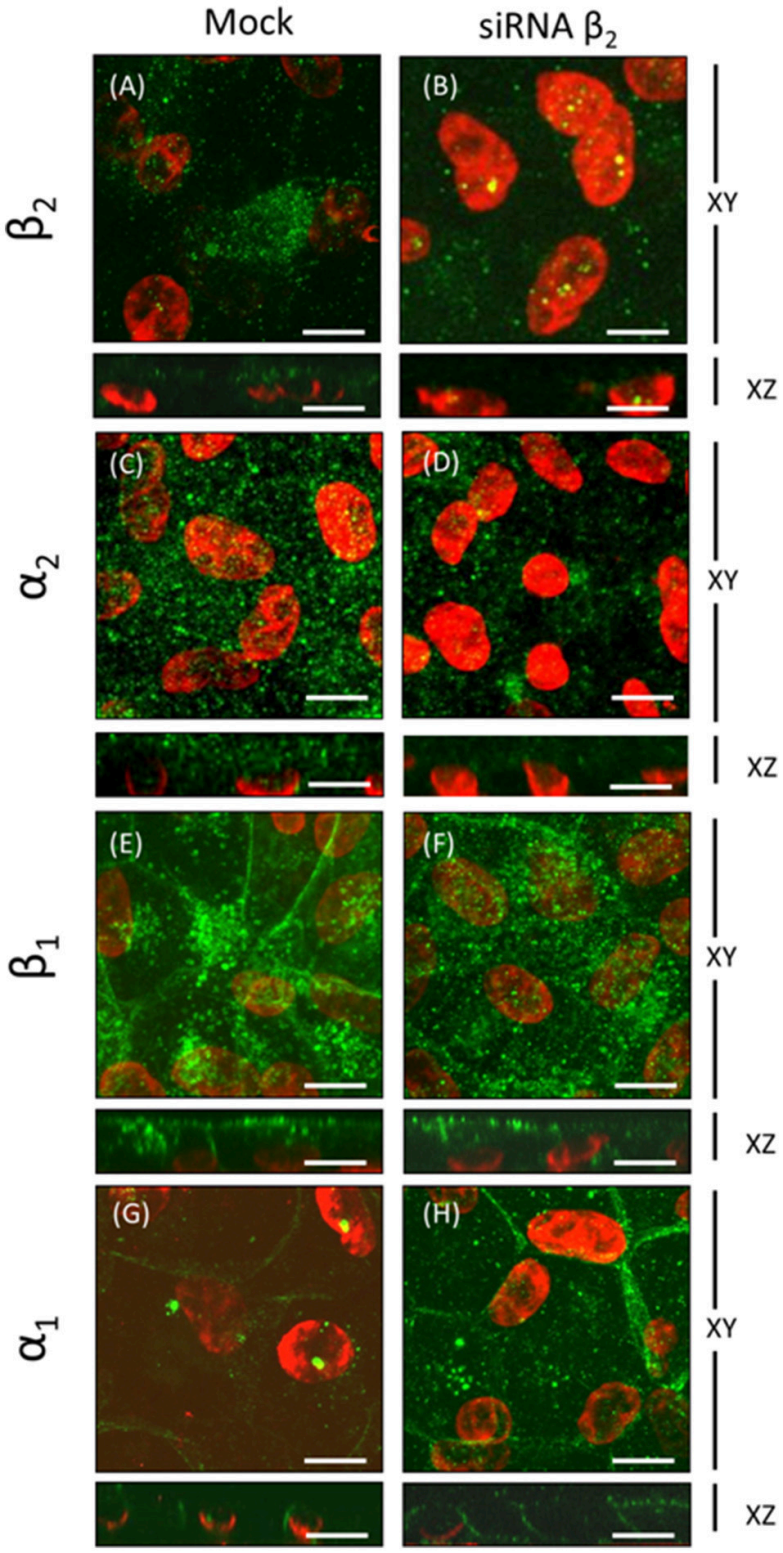
**Immunofluorescence analyses of ARPE-19 cells silenced with siRNA against the human β_2_ isoform**. ARPE-19 cells cultured for 4 weeks were transfected with or without siRNA specific to the human β_2_ isoform, as shown in Figure 8. Confocal images of silenced (right panels) or not-silenced (left panels) cells immunostained for specific α and β isoforms are displayed. Apical expression of the β_2_ and α_2_ isoforms in mock-transfected cells is apparent in **(A,C)**. Mislocalization and reductions in the fluorescence intensity of both β_2_ and α_2_ subunits are observed in silenced cells **(B,D)**. Basolateral and apical expression of the β_1_ isoform **(E)** is generally maintained in β_2_-silenced cells. However, in the presented field, β_1_ has a noted apical distribution pattern **(F)**. The mainly basolateral pattern of the α_1_ isoform **(G,H)** is maintained in silenced cells. Scale bar: 10 μm.

**Figure 11:**
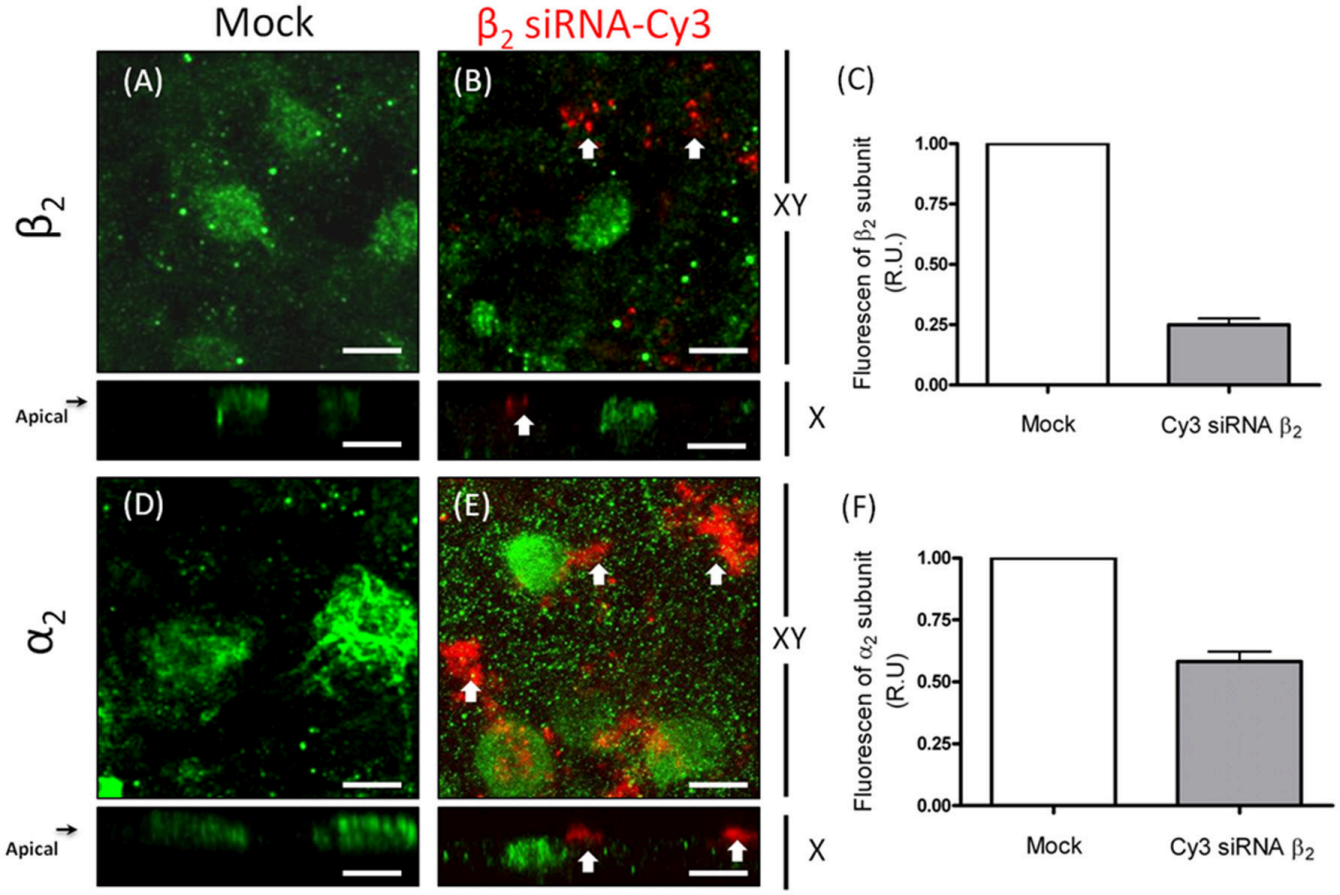
**Silencing of the β_**2**_ subunit in ARPE-19 cells affects the apical distribution of Na^**+**^, K^**+**^-ATPase**. The transfected siRNAs for the β_2_ isoform are labeled with Cy3. Red fluorescent spots thus identify transfected cells. Confocal images of mock-transfected cells in which the β_2_ and α_2_ subunits are immunostained **(A,D)**, respectively show an apical distribution pattern for both subunits. Images of silenced cells show an apical expression of the β_2_ **(B)** and α_2_ **(E)** subunits (green) in non-transfected cells. Arrows in **(B)** indicate the lack of β_2_ expression in the apical domain of siRNA-transfected cells. The absence of α_2_ immunostaining in the apical domain is also indicated by arrows in panel **(E)**, implying the mislocalization of Na^+^, K^+^-ATPase from the apical domain. The fluorescence intensity of β_2_ **(C)** and α_2_ subunits **(F)** in silenced cells was measured in 10 different fields, and the mean ± SEM is shown. Scale bar: 10 μm.

To further examine the dependence of the apical sorting of the Na^+^ pump on the expression of the β_2_ isoform, we knocked down the β_2_ isoform using siRNAs. As shown in Figure 9A, the expression of β_2_ mRNA in ARPE-19 cells treated with siRNAs specific for human β_2_ decreased by 60%. Meanwhile, the relative amount of α_2_ mRNA was also reduced. Nevertheless, the mRNA levels of the α_1_ and β_1_ isoforms were sustained at the same levels observed in the non-silenced cells (mock). The WB results for the total cell lysates (Figure 9B) show that the overall amount of β_2_ in siRNA-treated monolayers of ARPE-19 cells was only slightly decreased. This can be explained by the fact that ARPE-19 monolayers have a low index of proliferation and therefore a low rate of protein recycling. Although the amount of mRNAs is significantly reduced there is always a high amount of remnant proteins in the cells. Nevertheless, the IF images in Figure 10B show areas in the monolayer in which silencing was apparently effective because the fluorescence signal due to β_2_ expression in green was faint. At the same time, the apical expression of the α_2_ subunit (Figure 10D) also seemed to be reduced. The localization of β_1_ subunits changed substantially, displaying more apical rather than lateral distribution (Figures 10E,F). The lateral distribution of α_1_ subunit, as shown in Figures 6D,F, 10G, was also altered, showing a mixed distribution in the apical and lateral domains. The WB and IF results in Figures 9, 10 show a partial silencing. The siRNA-transfected monolayer still contained cells expressing the α_2_ and β_2_ subunits at the apical membrane, Therefore, it was difficult to determine whether the apical localization of the pump was indeed altered. Hence, we knocked down the monolayer with siRNAs that were pre-labeled with the fluorochrome Cy3 because it helped us identify probable silenced cells in a partially silenced monolayer. As shown in Figures 11A,B, the monolayer was not completely silenced, and β_2_-subunit staining was still observed at the apical domain in non-silenced cells (green fluorescence spots). Nevertheless, as shown in Figure 11B, expression of the β_2_ subunit in silenced cells that were stained with Cy3 (in red) is clearly weaker (white arrows), suggesting effective silencing in these cells. In Figure 11C, we measured the green fluorescence intensity of the β_2_ subunit in Cy3-positive cells. The average expression in these cells is reduced by 75%, indicating effective silencing. Expression of the α_2_ subunit was also monitored in silenced cells. As illustrated in Figures 11D,E, the apical localization of the α_2_ subunit was conserved. Nevertheless, measuring the fluorescence intensity of the α_2_ subunit in Cy3-positive cells revealed a 40% decrease in α_2_ subunit expression (Figure 11F). Our silencing results support the notion that the apical sorting of Na^+^, K^+^-ATPase in ARPE-19 cells during re-morphogenesis depends on the expression of the β_2_ isoform and its association with the α_2_ subunit.

## Discussion

RPE cultures appear to have a limited ability to reiterate epithelialization and undergo phenotypic maturation, a process described as re-morphogenesis by Burke (2008). In the present study, we first had to establish conditions that supported the re-morphogenesis of ARPE-19 cells *in vitro*. The appearance of Na^+^, K^+^-ATPase in the apical domain of ARPE-19 cells reflects the maturation and differentiation of the monolayer (Burke et al., 2000; Kannan et al., 2006; Sonoda et al., 2010). In this study, we addressed one aspect of the apical polarization mechanism: identifying the isoform that may contain the apical information (signal) necessary for the apical sorting of Na^+^, K^+^-ATPase in the RPE. Our observations indicate that the α_2_ and β_2_ isoforms are rarely detected in non-mature ARPE-19 cells (Figure 2) and constitute the apical pump in polarized RPE cells (Figure 8). We showed that the apical sorting of Na^+^, K^+^-ATPase in ARPE-19 cells correlates with the expression of the β_2_ subunit (Figure 6), a finding that is consistent with the observations in fixed eye sections (Figure 1). We also showed that during the process of re-morphogenesis, the expression of the α_2_ and β_2_ isoforms was up regulated (Figure 4) and that Sp1 is probably involved in that regulation (Figure 5). Although in the eye, the pumps composed of α_2_ and β_2_ subunits were localized in the apical membrane domain (Figure 8), they did not accumulate in the apical membrane in cultured ARPE-19 cells (Figure 7) but were probably retained in a sub-apical compartment. The increase in mRNA was much more pronounced than that of the extracted protein. Accordingly, silencing of the Na^+^, K^+^-ATPase β_2_ subunit by siRNA resulted in a decrease in the apical localization of Na^+^, K^+^-ATPase in knocked-down cells (Figures 10, 11) but without a clear change in α_1_/β_1_ localization.

### ARPE-19 cells as a model for studying polarity in the RPE

The RPE forms the outer blood-retinal barrier that regulates the movement of solutes between the capillaries of the choroid and the photoreceptor layer of the retina. Although human fetal RPE (hfRPE) primary cultures are considered the best model for exploring the polarity and trafficking mechanisms in RPE (Lehmann et al., 2014), we have no access to primary cultures of hfRPE. Therefore, we used the cell line ARPE-19, which was obtained from a spontaneously transformed human RPE primary culture (Dunn et al., 1996). ARPE-19 shows acceptable conservation of polarity and barrier function for studies of protein trafficking. The main advantages of ARPE-19 cells are its normal karyotype, relatively fast proliferation rate, and maintenance of several RPE-specific characters (Dunn et al., 1996). Lehmann et al. (2014) mention that in ARPE-19 cells, “the trafficking machinery is likely different from RPE *in situ* because the Na^+^, K^+^-ATPase was reported to be basolateral in ARPE-19 cells.” Based on our experiments, we suggest using greater precision when considering Na^+^, K^+^-ATPase polarity and discussing specific dimer compositions: α_1_β_1_ or α_2_β_2_. Thus, our data are consistent with the findings of Ahmado et al. (2011) with respect to the basolateral distribution of α_1_β_1_. Surprisingly, several studies do report an apical localization of the Na^+^ pump when using anti-α_1_ antibodies in ARPE-19 cells. Nevertheless, different authors define distinct patterns of localization based on IF images as apical (Geisen et al., 2006; Kannan et al., 2006). It is well documented that both primary cultures and cell lines tend to lose the RPE-specific properties with consecutive passages. The disruption of cell-cell adhesion induces an EMT, resulting in a loss of the RPE phenotype that can become irreversible (Grisanti and Guidry, 1995; Gallagher-Colombo et al., 2010; Tamiya et al., 2010; Adijanto et al., 2012). Accordingly, we suggest that α_1_β_1_ is the default dimer expressed and is sorted primarily to the basolateral membrane domain in non-differentiated ARPE-19 cells. During re-morphogenesis, only some ARPE-19 cells epithelialize to achieve a RPE phenotype, while others remain in a mesenchymal state. Here, we applied culture conditions that augmented the proportion of well-differentiated cells but still failed to obtain a fully differentiated cell population. Under these improved conditions, the expression of the α_2_β_2_ dimer was up-regulated, and after 4 weeks, there was a large proportion of cells with this dimer localized in a pattern resembling an apical distribution. Evidently, the α_2_β_2_ dimer was absent from the basolateral domain. The apparent apical localization probably depends on the maturation and differentiation of the apical trafficking machinery, which was also only partially achieved.

### The transcription factor Sp1 expressed in ARPE-19 cells is probably involved in regulating the expression of the β_2_-subunit

During re-morphogenesis, the mRNA and protein expressions of the α_2_ and β_2_ isoforms are up-regulated. It is conceivable that this long-range up-regulation suggests transcriptional regulation and thus the participation of transcription factors. Shull et al. (1989) and Ikeda et al. (1993) observed that Sp1 also activates the α_2_ promoter in rat and human skeletal myoblasts. Together, these data suggest that the transcription factor Sp1 is involved in the up-regulation of α_2_ and β_2_. Our observations (Figure 5) support these previous findings. Recent evidence points to a role for Sp1 in regulating the transcription of genes in response to extracellular signals such as insulin (Therien and Blostein, 2000). Hence, the addition of insulin (a component of the ITS mixture) to the culture medium could activate Sp1, promoting Na^+^, K^+^-ATPase expression via binding to positive regulatory *cis*-acting elements on the Na^+^, K^+^-ATPase β_2_ gene (Takeyasu and Kawakami, 1989; Sweadner et al., 2000; Tanos and Rodriguez-Boulan, 2008). Nevertheless, additional experiments are needed to clarify the mechanism of β_2_ regulation via Sp1 in ARPE-19 cells.

### The apparent apical polarization of the α_1_β_1_ and α_2_β_2_ dimers in ARPE-19 cultures

In classic epithelia, the mechanism underlying the basolateral polarization of Na^+^, K^+^-ATPase is related to the expression of the α_1_ and β_1_ subunits. Nevertheless, published reports on the isomer-specific composition of Na^+^, K^+^-ATPase in the RPE are somewhat confusing. Because the RPE originates from the neuroepithelium of the optic vesicle, we hypothesized that it would express the neuronal AMOG/β_2_ isoform, which was supported by our IF experiments depicted in Figures 1, 8. Nonetheless, the literature includes both consistent and contradictory reports. (a) An analysis of human RPE mRNA revealed the expression of the α_1_, β_1_ and β_2_ isoforms but not the α_2_ isoform (Ruiz et al., 1995, 1996). (b) The distribution of all subunits examined revealed that α_1_ and β_1_ were the predominant isoforms expressed in mouse and rat RPE, while the β_2_ isoform was detected in photoreceptors, bipolar cells and Müller glia but not in the RPE (Wetzel et al., 1999). (c) Most studies using RPE cells *in vitro* have utilized anti-α_1_ and anti-β_1_ antibodies for immunodetection of Na^+^, K^+^-ATPase (Miller and Steinberg, 1979; Rizzolo and Zhou, 1995; Burke et al., 2000; Kannan et al., 2006). Our observations suggest that in non-polarized ARPE-19 cells, the ubiquitous α_1_β_1_ dimer is the default housekeeping Na^+^ pump essential for all living cells. This dimer likely uses the non-differentiated trafficking mechanism to arrive at the plasma membrane and is then stabilized and enriched in cell-cell contacts due to β_1_-β_1_ *trans* interactions between neighboring cells. Considering that the β_2_ subunit is an adhesion molecule, in the eye, it would interact with a heterotypic adhesion protein localized on the outer segment of the photoreceptor membrane, maintaining the complex at the apical domain. Our images taken from human eye sections (Figure 8) support this assumption. Accordingly, it is plausible that Na^+^, K^+^-ATPase is only detected at the apical domain of cultured RPE cells under very specific conditions (Hu et al., 1994; Marrs et al., 1995; Rizzolo and Zhou, 1995; Kannan et al., 2006; Sonoda et al., 2010) because of the lack of an interaction of RPE cells with photoreceptors in cultures. However, in various RPE models, Na^+^, K^+^-ATPase is observed in the apical domain, even in the absence of contact with the retina. Nevertheless, no previous studies have confirmed these observations via co-staining with apical markers or surface biotinylation. Evidently, the absence of photoreceptors on the apical side of cultured RPE cells does not mean that the Na^+^ pump is not being sorted and delivered to that domain, but it implies that the α_1_β_1_ dimer, observed mostly using anti-α_1_ antibody, is actually a non-polarized pump that is directed to all membrane domains, including the apical one (Figure 6 and Hu et al., 1994; Sonoda et al., 2010; Kannan et al., 2006). Our silencing experiments support this notion, as silencing the β_2_ isoform diminished the apical localization of theα_2_β_2_ dimer but not that of α_1_β_1_ (Figure 10).

### Apical/basolateral sorting of multimeric membrane proteins is an intricate mechanism

**Figure 12:**
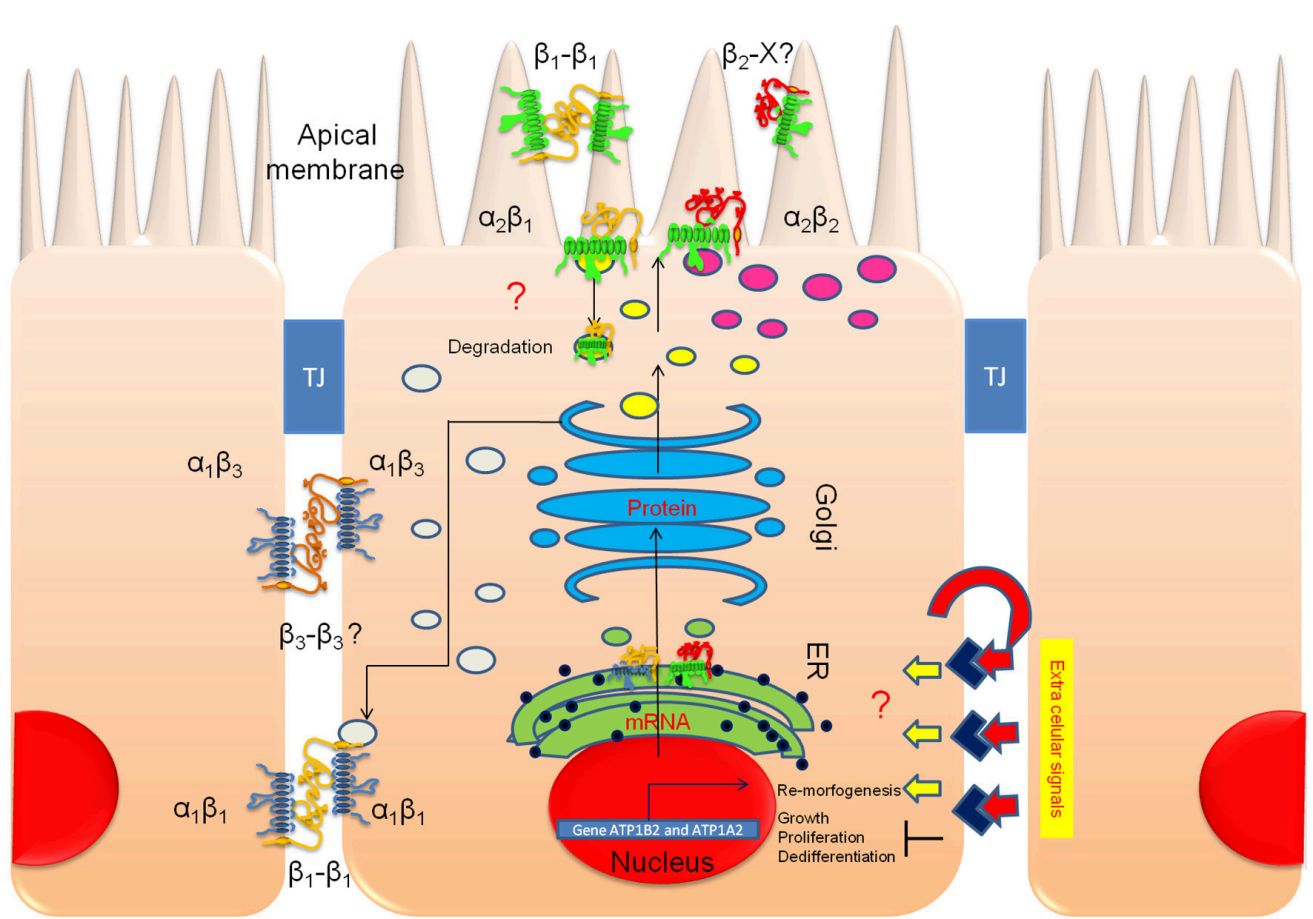
**The apical polarization of Na^**+**^, K^**+**^-ATPase in polarized ARPE-19 cells is regulated by the β_2_ subunit**. An illustration of polarized ARPE-19 cells cultured for 4 weeks on permeable inserts in the presence of ITS is depicted; the cells are relatively tall, express RPE markers and form adjacent tight junctions (TJs). Our model proposes that extracellular signals trigger transduction pathways that activate re-morphogenesis. In non-polarized ARPE-19 cells, a basolateral targeting mechanism carries α_1_β_1_ and α_1_β_3_ dimers to the lateral membrane (gray vesicles). The β_1_-β_1_ (and perhaps β_3_-β_3_) trans-interaction between neighboring cells stabilizes and retains these dimers at the lateral membrane domain for housekeeping. Upon the triggering of re-morphogenesis, a concurrent apical targeting mechanism is activated by the association of the α_2_ and β_2_ subunits. α_2_β_2_ is delivered to the apical domain (magenta vesicles). In cultures, this complex probably does not stabilize at the apical plasma membrane but accumulates in a sub-apical compartment. *In situ*, the pump is inserted in the apical membrane domain, where it is probably stabilized by heterotypic trans interaction(s) of the β_2_ subunit with adhesion proteins on the outer segments of the photoreceptor (β_2_-X).

Current models of apical/basolateral sorting mechanisms in epithelia are mostly based on evidence obtained for monomeric proteins (TfR, LDLR, and FcR; Matter et al., 1994; Gan et al., 2002; Perez Bay et al., 2014). Corresponding models for multimeric proteins, such as Na^+^, K^+^-ATPase, have not been clearly established. It is accepted that polarized proteins carry apical or basolateral sorting signals and that in some cases, two or more opposing signals co-exist in the same protein (Philp et al., 2011). In the case of Na^+^, K^+^-ATPase, the α_1_ subunit contains an unidentified dominant basolateral signal. However, it has been established that the β subunits also contain sorting information that is recessive relative to the basolateral signal of α_1_. In particular, the N-glycosylation of the β_2_ subunit functions as an apical sorting signal (Vagin et al., 2005). As shown by Castorino et al. (2011) for the sorting signals of CD147, the same sorting signal can be interpreted in different ways in distinct cell types. This observation justifies investigating the role of the apical sorting signal of the β_2_ subunit in ARPE cells. Our present observations in polarized ARPE-19 cells, summarized in Figure 12, partially clarify some of the complicated and confusing data presented in the literature, as illustrated by the following points. (a) Na^+^, K^+^-ATPase evaluated based on the expression of the β_1_ isoform was detected in both the basolateral and apical domains, consistent with the findings of Hu et al. (1994). (b) The predominant combination at the apical membrane domain of polarized ARPE-19 cells is the α_2_β_2_ combination, which is consistent with the concept that the α_2_ isoform is the preferred binding partner of the β_2_ isoform in assembling the Na^+^, K^+^-ATPase in different tissues (Lin et al., 2005; Harada et al., 2006; Tokhtaeva et al., 2012) and with the co-localization of α_2_β_2_ in human eye sections shown in this work. (c) Polarized ARPE-19 monolayers express Na^+^, K^+^-ATPase subunits in a membrane domain-specific pattern: α_1_ is detected only at the basolateral domain, α_2_ is present only in the apical domain (as seen in Figure 6), β_1_ subunits are localized in both the basolateral and apical domains, β_2_ subunits are preferentially localized in the apical domain, and β_3_ subunits are exclusively localized in the basolateral domain. Together, these results suggest that α_1_β_1_ and α_1_β_3_ are the basolateral combinations. It is unclear whether apical β_1_ is a mislocalized α_1_β_1_ dimer or a non-preferential combination with α_2_. It is most likely an α_2_β_1_ dimer because we did not detect α_1_ subunits in the apical domain (Figure 4). (d) IF assays in human eye sections (Figures 1, 8) reveal apical staining of the RPE using antibodies specific to α_2_ and β_2_ isoforms. These data indicate that the apical Na^+^, K^+^-ATPase in RPE cells includes the α_2_β_2_ dimer. Thus, a comprehensive analysis of the sorting machinery and trafficking routes that direct the α_2_β_2_ complex to the apical domain in polarized ARPE-19 cells must be performed in future studies.

## Author contributions

JL designed and performed the experiments, analyzed the data, made the figures and wrote the manuscript. JB, MR, and TL performed experiments. RG and JB designed the experiments and revised the manuscript. LS designed the study, analyzed the data and wrote the manuscript.

## Funding

This study was supported by a grant for LS and a Doctoral scholarship for JL and TL from CONACYT-MEXICO.

### Conflict of interest statement

The authors declare that the research was conducted in the absence of any commercial or financial relationships that could be construed as a potential conflict of interest.

## Abbreviations

ITS (mixture of insulin transferrin and selenic acid): RPE (retinal pigment epithelium).
